# Closer to or further from the new normal? business approach through social media analysis

**DOI:** 10.1016/j.heliyon.2021.e07106

**Published:** 2021-05-25

**Authors:** Patricia P. Iglesias-Sánchez, Carmen Jambrino-Maldonado, Carlos de las Heras-Pedrosa, Elena Fernandez-Díaz

**Affiliations:** a Department of Economics and Business Organization, Faculty of Economics and Business Studies, Universidad de Málaga, 29071, Malaga, Spain; b Department of Audiovisual Communication and Advertising, Faculty of Communication Sciences, Universidad de Málaga, 29071, Malaga, Spain

**Keywords:** COVID-19, Social media, Business, Economic effect, New normal

## Abstract

The outbreak of COVID-19 has led to radical change in all social and economic spheres and, even today, the scope of the pandemic cannot be detailed. This unprecedent situation is challenging the global world but particularly for business. The packages of measures internationally imposed as restrictions on commercial activity, isolation and social distancing mean that business should face a transformation in order to survive in each stage of the crisis. For this purpose, a content analysis with an initial dataset with 2,610 tweets of the most representative Spanish entrepreneurial organizations was carried out in key periods of the pandemic. The findings highlight that there are collective concerns with emotional burden in the business sector that encourage action despite confusion and uncertainty. Generalized distrust of policies led business organizations to insist on innovation and adaptation as the best tools to overcome the economic effect of the crisis.

## 1 Introduction

Daily life and economic activity have radically changed due to COVID-19. This respiratory disease, which started in Wuhan (China) at the end of 2019, has resulted in the most severe disruption of the global economy since World War II (Gössling et al., 2021). The health crisis, declared pandemic by the World Health Organization (WHO) in March 2020, highlighted not only social consequences due to the death toll but also speed of contagion and has affected international economic activity. Even today, the evolution of the disease and its economic impact is highly uncertain (McKibbin and Roshen, 2020) and raises new challenges for business and policymakers. It is an unprecedent situation that implies the introduction of disruptive and severe measures to contain the pandemic, including temporary cessation or restriction of economic activity, isolation and social distancing. According to Gourinchas (2020) this disaster has caused a cascading chain of disruptions, including market volatility, reduction of household expenses, labour market shock, and an increase in prices amongst other things. However, what is more important, the indirect effect post-health crisis, for example, the direct loss of Gross Domestic Product (GDP) will be largely reflected in a decline in the consumption of goods and services or the fall in output will lead to a significant reduction in employment and, consequently, a loss of income and consumption (Galí, 2020). Precisely, the impact beyond public health introduces concepts such as syndemic by Singer and Clair (2003) and becomes important in the current situation due to the need to provide a holistic approach not only focused on clinical medicine and public health. COVID-19 impact demands analysis from different points of view because the resolution of the crisis is not restricted to control of the disease. Without any certainty about the end of the crisis, the challenge is to achieve the “new normal” and for entrepreneurs this means reopening and recovering initiatives and activity (Samuel et al., 2020).

Several early extensive pieces of literature are about the economic impact of COVID-19 (Atkeson, 2020; Baldwing & Weber Di Mauro, 2020; Bartik et al., 2020) and sentiment and topics analysis derived from this health crisis (Abd-Alrazaq et al., 2020; Buckman et al., 2020; de las Heras-Pedrosa et al., 2020a, b). However, there are very few studies which focus on the sentiment of economic impact and uncertainty/challenging situations that companies are facing at present and in the immediate future (Bartik et al., 2020; Fetzer et al., 2020; Samuel et al., 2020).

This study aims to analyse how entrepreneurial organizations manage this unknown scenario for business through their communication in social media. These kinds of organizations represent the business community and, in a certain way, express the collective concern and provide a new framework where common challenges for the “new normal” can be addressed. The content generated in the three most representative business organizations in Spain shows the current disaster has led to escalating emotional and mental health issues (Goldmann and Galea, 2014; Samuel et al., 2020) for entrepreneurs and how uncertainty, distrust and fear condition the response to re-activate and return to economic activity in a completely new framework for everyone. Moreover, this research work presents the particular case of Spain, one of the most affected countries in Europe.

This paper is divided into five sections. After this introduction, we focus on the recent literature about the economic effect of COVID-19. Next, method is explained. A qualitative technique is carried out based on analysis of the content of the official accounts of the most representative organizations linked with enterprises. The fourth section explains the empirical component and, the discussion and conclusion section determine how innovation and the search for scenarios and tools is firmly committed to overcoming the devastating consequences for business. Finally, this research work presents practical implications for policy makers.

## 2 Theoretical framework

### 2.1 Economic effect of COVID-19

Barua (2020) summarizes economy macroeconomic shocks of the pandemic on demand, supply, supply chains, trade, investment, price levels, exchange rates, financial stability and risk, economic growth and international cooperation. Furthermore, Baker et al. (2020) stress that the extent to which the pandemic has effected consumer behaviour, business survival, unemployment rates, R&D, human capital investment etc and all of them affect productivity negatively over the medium and long term. According to several analyses, the most dramatic effect is not directly linked with the disease but with uncertainty. In fact, Wren-lewis (Wren-Lewis, 2020) estimates that the direct impact of the pandemic on GDP is a few percentage points because this depends on the proportion of the population that gets sick and dies but the figures increase if dismissals, closed businesses and interruption or decreasing of entrepreneurial activity are included. Furthermore, this author highlights that school closures amplify the reduction in labour supply and can multiply the impact on GDP. As result of this, the uncertainty about the future of the pandemic in all spheres grows and, thus, economic consequences show a negative trend (United Nations Conference on Trade and Development, 2020). For example, Organization for Economic Cooperation and Development (OECD) (OECD, 2020) anticipates a rise of only 10 base points in investment risk in all countries and increases the cost of capital and reduces investment dramatically due to the high level of uncertainty. Additionally, the severe measures of containment of the disease promote fear and confusion in the general population and business that directly impacts on cut back investments and consumption (Baldwing and Weber Di Mauro, 2020). As result of the outbreak of the pandemic – and it is not over yet - businesses closed, were forced to change their business models radically or eliminate tasks that will not bring value in the current environment, which have reached critical rates (Donthu and Gustafsson, 2020). In contrast, authors such as Hassan et al. (2020) pay attention to opportunities that could emerge in specialized activities such as pharmaceutical, medical devices, protection and Occupational Health and Safety (OHS) or Information and Communication Technology's (ICT) activities because the crisis has brought about a supposed digital revolution as well. Consequently, these authors introduce *losers* and *winners* of the COVID-19 outbreak.

In any event, economic activity has to re-start, workers have go back to work… (Gourinchas, 2020). Thus, we need to answer how businesses perceive and react to the challenges of the “new normal”, in this case through communications in social media made by the most representative organizations for entrepreneurs. Also, how uncertainty conditions their strategies and decisions to turn back on. We can even examine if these organizations encourage businesses find an opportunity in this crisis or, by contrast, do all activities feel like losers focusing on criticism and dramatic messages due the outbreak. These questions are answered focusing on conversations in social media.

### 2.2 Business concerns in relation to the pandemic

This unprecedent pandemic has brought disturbing changes from all directions even complex challenges in lifestyle for people and in dynamics/routines for companies. The adverse circumstances have led to extreme feelings, and emotional and mental healthcare challenges (Samuel et al., 2020). The effect of disaster on mental health has already been addressed and theorized by Goldman and Galea (Goldmann and Galea, 2014) but, maybe the novel coronavirus has been a particularly suitable framework to test it. The mortality, the speed of contagion and the application of the global restrictions in economic activity, stay at home measures with long isolation periods, the extent of social distancing and the imperative use of masks, amongst other things, generate stress and imply radically changing the way of organising and understanding the world. Examples of the dimension of this challenge are teleworking, e-education and, even, the de-globalization phenomenon (Barua, 2020). Moreover, the direct consequences in companies shutdown, reduction in staff or number of work centers, radical restructuring of activities, required investment and implementation of established measures of COVID-19 contention generate an irretrievably deep consternation in the business sector that will not be solved in a short time Donthu and Gustafsson (2020); Baldwing & Weber Di Mauro (2020). As result of this, the decisions and strategies adopted are shaped by a special framework to make economic activity more difficult.

Recent research work such as Samuel et al. (2020) highlights extreme fear, confusion and volatile sentiments, while Iglesias-Sánchez et al. (2020) stress the coexistence with peaks of joy, sadness, anger, fear and disgust. Likewise, Mann (2020) shows that all sentiments have a negative component during the crisis through authorities, consumers and business perception. Nevertheless, the studies focused on sentiments derived from COVID-19 agree that uncertainty is the predominant and most reiterative and, in a certain way, it affects emotional, mental and behavioural reaction. It could be useful to discover the collective sentiment trends for decision makers, both of politicals leaders and entrepreneurs, in order to overcome this crisis (McKibbin and Roshen, 2020). Furthermore, corporate resilience is identified as a consequence of the spread of the respiratory disease (Hassan et al., 2020) and it can be seen as a direct response to the outbreak in order to mitigate and reinvent business (Gourinchas, 2020; Samuel et al., 2020). Until now, research work in this stream has specifically focused on citizens but the treatment of the issue from the business point of view is scarce. Moreover, in the current circumstances of the pandemic, the analysis of shared concerns and the influence of a collective awareness of how to face this problem seems to be a direction of merit for consideration by researchers and practitioners.

This is the reason the emphasis is placed on analysing thoughts, expectations and sentiments from business point of view, especially regarding the challenge of the “new normal”. The COVID-19 catastrophe is producing large scale, global and persistent economic disruption (Baldwing & Weber Di Mauro, 2020), the effect on sentiments that influence the reactions and predisposition to face the recovery in the social and economic sphere. For this reason, this study understands all types of business as entrepreneurs: self-employed and companies independently of their size, assume risk and carry out an economic activity. This matches the definition given by OCDE (1997) that state the entrepreneur is –irrespective of the legal form-the person or persons who develop the dynamic process of identifying economic opportunities and acting upon them by developing, producing and selling goods and services.

As a result of this, the following research questions are posed: what are the entrepreneurs’ collective concern and sentiment about the future of economic activity, reopening scenarios and strategies to succeed the “new normal”? How far are these thoughts and feelings seized upon to design strategies and react to the current crisis, in the case of Spanish business? It should be empathized that this focus is analysed through the collective business interest and voice that brought together the business organizations that represent them. This idea is previously supported by Pandey and Gupta (2008) and Eldor (2020) among others.

### 2.3 Crisis through social media

Crises generate ambiguity and uncertainty, so it is important to transmit information to stakeholders (DiStaso et al., 2015). Currently, the dissemination channels have extended and social media play a main role (Cheng, 2018; Coombs, 2007; Eriksson, 2018; Roberts and Veil, 2016; Rodin et al., 2019; Tække, 2017). Online conversations focused on the pandemic have been growing and spreading at an instantaneous speed from the beginning of the crisis (de las Heras-Pedrosa et al., 2020a; De las Heras-Pedrosa et al., 2020b; Han et al., 2020; Nazir et al., 2020). Social media are not only a source of information, they enable sharing and reach millions of people (Maal and Wilson-North, 2019). According to Abd-Alrazaq et al. (2020) open and global access to content generated on social media platforms can be used to identify the main thoughts, attitudes, feelings and topics about COVID-19 that are on people's minds. Thus, Fetzner et al. (Fetzer et al., 2020) show that social media influences the public's perceptions and even economic expectations in times of the pandemic. In spite of this fact, this issue has been pointed out as a result of the high uncertainty linked with crisis (Prat and Strömberg, 2013) the current crisis has made this especially clear (Baker et al., 2020; Buckman et al., 2020). This allows us to suggest that the analysis of entrepreneurs' conversations on social media can be an excellent platform to better know how they perceive and face the challenge of the “new normal”. Furthermore, we respond to whether social media reflects a collective concern and if common strategies and reactions emerge as a result of it. Moreover, this analysis can support the crisis responses as is suggested by Wang et al. (2020). In this case, business organizations are understood to be authorities and leaders for expressing collective entrepreneurial concerns and the support for defining directions and actions in the corporate sector.

## 3 Material and methods

### 3.1 Sample

The research methods are based on the observation on Twitter through content analysis and comparative metrics between the three most representative official accounts of organizations focused on entrepreneurs in Spain: Spanish Chamber of Commerce (@camarascomercio), Confederation of Employers and Industries of Spain (CEOE) (@CEOE_ES) and National Federation of Associations of self-employed workers (@autonomosata) Table 1. Restricting the study to one country, Spain, is especially interesting because of the dimension of the pandemic due to the death toll, the contagion and the devastating consequences on the structure of its economy as well.

**Table 1:** Role and social media impact of analysed organizations.

Organization	Description and role	Social media presence	Twitter followers^1^	Tweets volume^2^
Spanish Chamber of Commerce @camarascomercio	Spanish Chamber of Commerce is a public law corporation whose purpose is the representation, promotion and defense of the general interest of Spanish companies.	Twitter Facebook Youtube Linkedin	23.5 mil	58.4 mil tweets
Confederation of Employers and Industries of Spain (CEOE) @ceoe_es	CEOE is one of the most representative employers' organizations in Europe. It brings together, ​on a voluntary basis, more than 1,200,000 companies from the whole of Spain, covering all ​sectors of the economy.	Twitter Facebook Youtube Linkedin Instagram Flickr	30 mil	19.6 mil tweets
National Federation of Associations of self-employees (ATA) @autonomosata	The National Federation of Self-Employed Workers (ATA) has been defending and representing the self-employed for more than two decades. It is currently a nationwide organization made up of more than 210,000 freelance shareholders who, added to the transfer of representation, lead ATA to add more than 450,000 freelancers.	Twitter Facebook Youtube Instagram Linkedin Soundcloud	25.7 mil	30.6 mil tweets

The combined analysis of these three official accounts provides a holistic and integrated vision of business of economic impact. It was stated that the analysis of more than one case in the same study might yield more accurate results (Eisenhardt and Graebner, 2007).

### 3.2 Measures and instrumentss

The content analysis in the targeted Twitters account allows identifying economic sentiments and expectations about “new normal” from business point of view. The conversations generated in social media introduce possible scenarios, strategies, proposals, doubts, and criticism as well.

Under the considerations made by Weber (2020), content analysis makes it possible to draw inferences from social media postings. The comparison of three institutions allows the simultaneous study of collective concerns and challenges for entrepreneurs and the existence of corporate resilience in this context (Hassan et al., 2020) through the observable features of the written texts, images and videos embedded in the message. As stated above, recent and previous literature is mainly focused on economic effects but little attention is paid to effects of collective sentiments to reacting to a crisis like the pandemic derived from COVID-19 (Gourinchas, 2020; McKibbin and Roshen, 2020; Samuel et al., 2020). However, the approach has been carried out through the analysis of communications made by entrepreneurial organizations as key representatives of the business collective (Pandey and Gupta, 2008; Eldor, 2020) and not monitoring a sample of self-employed entrepreneurs or companies.

The design of the evaluation sheet for content analysis was based on work by several authors (Baldwing & Weber Di Mauro, 2020; Barua, 2020) regarding economic items. Additionally, some extra variables to introduce detailed information about each tweet (image, video, likes) have been added, as well as tone and resilience. As has been explained in the theoretical framework, corporate resilience is a direct response to the pandemic and it promotes the transforming of the business (Gourinchas, 2020; Samuel et al., 2020), as a result of this, it is a key issue to analyse in the paper. Finally, the content category and main theme are included. It is worth mentioning that this qualitative analysis uses variables and indicators that have been validated in previous studies, but the contribution is precisely their combination on the evaluation sheet, which provides a holistic and different vision, which is more focused on concerns and feelings of impact not specifically the metrics of the economic impact. In any event, it should be pointed out that the content analysis, according with Krippendorf (2009) is not strictly qualitative because some variables are coded with quantitative measurement (for example: followers, number of posts, likes, comments, retweets, tone and resilience). Moreover, the coding process allows the most significant results beyond a qualitative analysis to be shown. Consequently, this study results in a combination of a quantitative technique and qualitative technique. This choice provides us a better understanding of connections and importance regarding the main issues throughout the research process.

To sum up, this research work proposed eight blocks: I Tweets, II. Concerns about the economical effect of COVID-19, III. Shocks disrupting economy, IV. Proposals for reactivating economy/reopening, V. Tone, VI. Resilience, VII. Content Category, VIII. Main theme, Table 2.

**Table 2:** Monitoring analysis content sheet.

Tweets	Date	
	Text	
	Link	
	Image	
	Video	
	Likes	
Concerns about economical effect of COVID-19 (Barua, 2020)	Production shocks:	Interruption, problems and levels of production
		Supply chain
		Demand
		Human movements
	Effects on international trade and capital flows	
	Macroeconomics effects:	Aggregate supply shock
		Aggregate demand shock
		Shock to price level
		Effects on employment and income
		Effects on exchange rates
		Effects on financial stability and risk
		Economic growth effects and the looming recession
	Economic growth effects and the looming recession	
	Potential for shifts in international relations and economic cooperation	
	Household spending	
Shocks disrupting economy (Baldwing and Weder Di Mauro, 2020)	Import and export	
	Business bankruptcies	
	Labour market:	Unemployment
		Teleworking
	Digital economy	
	Fiscal policies	
Proposal for reactivating economy/reopening (Baldwing and Weder Di Mauro, 2020) Original Expectations about policies	Financial regulation policies	
	Monetary policies	
	Social insurance policies	
	Industry policies	
	Taxes relief	
	Trade policies	
	Subsidies	
Tone	5. Very Positive, 4. Positive, 3. Neutral, 2. Negative, 1. Very Negative	
Resilience	5. Very Resilience, 4. Resilience, 3. Somewhat resilience, 2. Little resilience, 1. Not at all	
Content category	1. Criticism	
	2. Proposal estrategy/action	
	3. Project/programme	
	4. Analysis of impact/Forecast	
	5. Information	
	6. Other	
	Award	
Main Theme	Restoring confidence	
	Reopening	
	Transforming	
	Adaptation to the new normal	
	Corporate message of support	
	Prospect of a return to normal	
	CSR	
	Success example	
	Acknowledge	
	Appeal for individual ​and collective responsibility	

It should be emphasized that the social media metrics widely referenced (Peters et al., 2013; Vatrapu et al., 2016) have been used to ascertain the targeted Twitter account and their influence and the impact of the most common entrepreneurs’ concerns as well. Furthermore, a correlation analysis and analysis of variance (ANOVA) is carried out to test relationships between different variables. The qualitative analysis carries far more weight than the statistical analysis but a general descriptive analysis is performed and the main correlations between key factors in this research are underlined. Consequently, it is included because it provides an extra value by a set of key factors and complements to the content analysis.

The complete content of @camarascomercio, @CEOE_ES and @autonomosata were analysed: written content, images, videos and even additional information linked with the post. Despite the fact the initial database was 2610 tweets, it was pre-processed to cleanse the data and select only the content published related with the COVID-19. The total were 600 posts: 339 from the Spanish Chamber of Commerce, 74 from the Confederation of Employers and Industries of Spain and 187 from the National Federation of Associations of self-employed workers (Table 4). It should be highlighted that 600 tweets were 100% of post directly related with COVID-19 during the analysis period.

### 3.3 Data collection

There are two reasons for focusing on Twitter. On one hand, it is rather high in crisis management analysis, due to the profile of content published on this social media. On the other hand, the editorial line in official accounts follows a common pattern despite the inherent differences between digital platforms. Furthermore, it was tested with a random sample of posts in the three Twitter accounts that the content is exactly the same in the digital ecosystem managed by each organization. As a result of this, it is possible to generalize the approach provided by Twitter in this case.

All chosen periods are representative of the coronavirus crisis, especially for their economic effects:

• From 14 ^th^ to 20 ^th^ March: Decree of a state of alarm
• From 31^st^ March to 6 ^th^ April: Declaration of suspension of non-basic activities
• From 9 ^th^ to 16 ^th^ May: Official date of entry into the “new normal” days (Phase 4)
• From 1^st^ to 7 ^th^ July: date of the first finalization of the employment regulation
• From 15 ^th^ to 21^st^ September: one week after the ending of the peak summer season, in order to analyse the impact of the “new normal” on travel.

### 3.4 Validity and reliability

Despite the qualitative approach, it is important to highlight the soundness of a methodological design with three cases. They are the main authorities in the economic field and show similarity in their service and their business representation. However, their distribution was not similar in terms of number of entries, interactions, followers, growth etc. (Table 3).

**Table 3:** Representation of COVID-19 tweets.

		Total post	Percentage post related COVID-19
ORGANIZATION	ATA	1484	13%
	Chamber of Commerce	891	38%
	CEOE	235	31%
Total		2610	23%

## 4 Results

The analysis has been carried out with 600 posts from the official Twitter of ATA, Spanish Chamber of Commerce and CEOE. The focus was on specific content generated, related to COVID-19 in those social media profiles eliminating retweets (Table 3). It highlights that the topic has received great attention continuously over the chosen key period of time. It is even true to say that as time goes by, the main and, almost exclusive, limelight of COVID-19 is reduced and the space is shared with other topics. For example, when the state of alarm was declared @camarasdecomercio published 57 tweets linked with the pandemic, while in the last analysed period (15/09–21/09) there were 35. Therefore, the average publications were 14 per day, although in the first and third waves, the tweets were higher, 18–20 per day. Table 1 shows the weight in the total text corpus for the three targeted organizations. Table 4 shows disaggregated total tweets by organization and waves.

**Table 4:** Total post by organization.

		Total tweets	14/03/20 - 20/03/20	31/03/20 - 06/04/20	09/05/20 - 15/05/20	01/07/20 - 07/07/20	15/09/20 - 21/09/20
			1st Wave	2nd Wave	3rd Wave	4th Wave	5th Wave
ORGANIZATION	ATA	187	27	68	34	13	45
	Chamber of Commerce	**339**	59	109	86	49	36
	CEOE	**74**	21	11	9	13	16
**Total Tweets**		**600**	**107**	**188**	**129**	**75**	**97**

It should be emphasized that almost all of the publications had an image (94%) and only 6% of contents a video. However, a correlation between tweets with video and a higher level of reaction (likes) or interaction (retweets and/or comments) is not detected. Moreover, most of the videos are the same, repeated over the time, one of them is even a resource in social media for two organizations, video of @Spainforsure.

**Figure 1:**
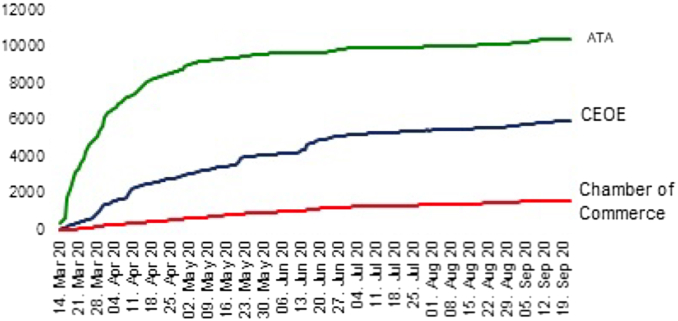
Followers' evolution.

Figure 1 and Table 5 underscore the trend of the increase in followers in the three accounts over the time of the study. The least representative growth was in @camarascomercio Twitter and the most eye-catching is associated with @autonomosata. In total, 80,471 people kept in touch with the social media of these institutions.

**Table 5:** Followers by organization.

		14/03/20 - 20/03/20	31/03/20 - 6/04/20	9/05/20 - 16/05/20	1/07/20 - 7/07/20	15/09/20 - 21/09/20
		1^a^ Waves	2^a^ Waves	3^a^ Waves	4^a^ Waves	5^a^ Ola
ORGANIZATION	ATA	19007	22962	25038	25615	26282
	Chamber of Commerce	22224	22521	22969	23451	23810
	CEOE	24991	26215	28002	29084	30649
Total		**66222**	**71698**	**76009**	**78150**	**80741**

Before detailing the published content on the three entrepreneurial organizations the communication strategies have been identified.

**Figure 2:**
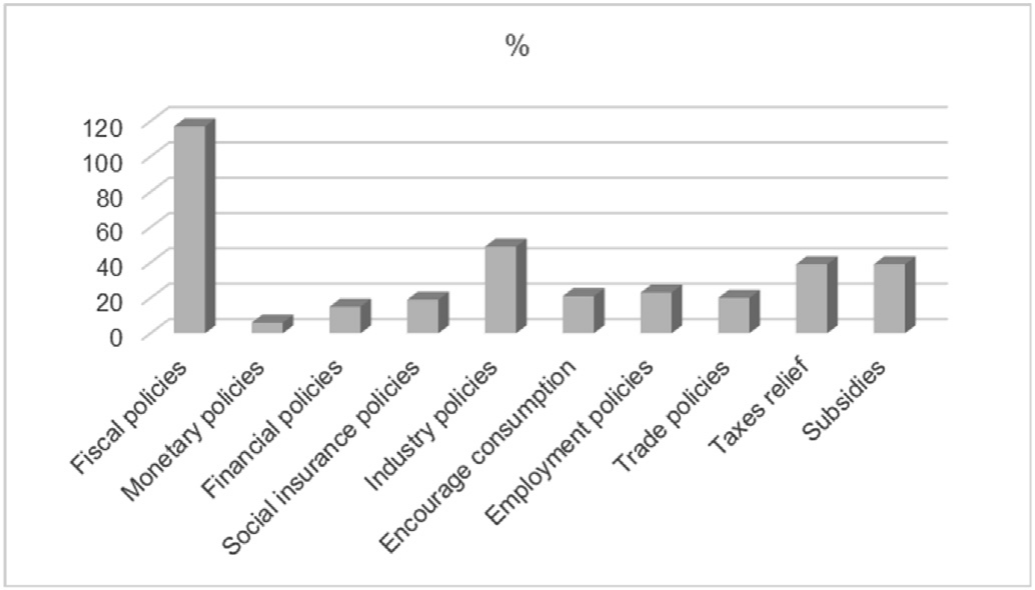
Total proposals for reactivating the economy.

All the organizations adopt the same strategy, they are focused on solutions and on calls to action to overcome the crisis. In the first phase, the information about measures and figures has more weight but, in any case, reaches the volume of post about initiatives, projects and recommendations to face the outcome (16% vs 72%). Direct criticism is not the common line for publishing on their social media for the whole period (10%). Figure 2 demonstrates that the official accounts of the three entrepreneurial institutions agreed on the proposals to overcome the pandemic from an economic point of view. The first position in the ranking of tweets is industrial policies followed by the request for subsidies for enterprises. The presence of the rest of the proposals is not so significant but the following should be emphasized: financial measurement, encouragement of consumption and tax relief. In any event, it is important to note that there are differences in the way of making the proposals. For example, @camarasdecomercio developed some of the proposals through their program and project while @autonomosata or @ceoe made specific requirements to the Government. The proposals were focused on reactivating the economy but specifically cushioning adverse effects of the pandemic on company performance and supporting them along the way to the “new normal”.

**Figure 3:**
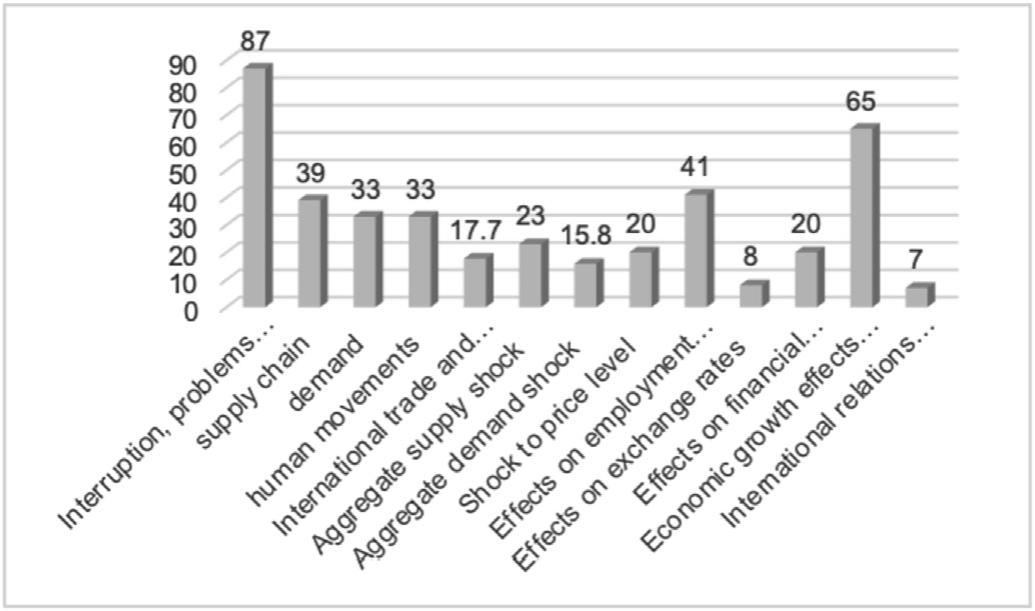
Concerns in tweets disaggregated.

The three institutions concentrate the most attention on production shock according to the CA. However, the most recurrent concern regarding production shocks is interruption of activities and problems and levels of production. This concern is followed by distance –but with a similar weightiness-by the supply chain problems, the decrease of demand and human movement linked with the pandemic (collective dismissal of workers, eduction of time worked, adaptation for teleworking etc). Macroeconomic effects highlight two concerns: economic growth effects and the looming recession and the effects on income and employment. By contrast, it should be emphasized that the issues least referred to on Twitter are linked with internationalisation. In fact, effects on exchange rates, effects on international trade and capital flows and the potential for shifts in international relations and economic cooperation were rarely related to them. The detailed information can be seen in Figure 3.

**Figure 4:**
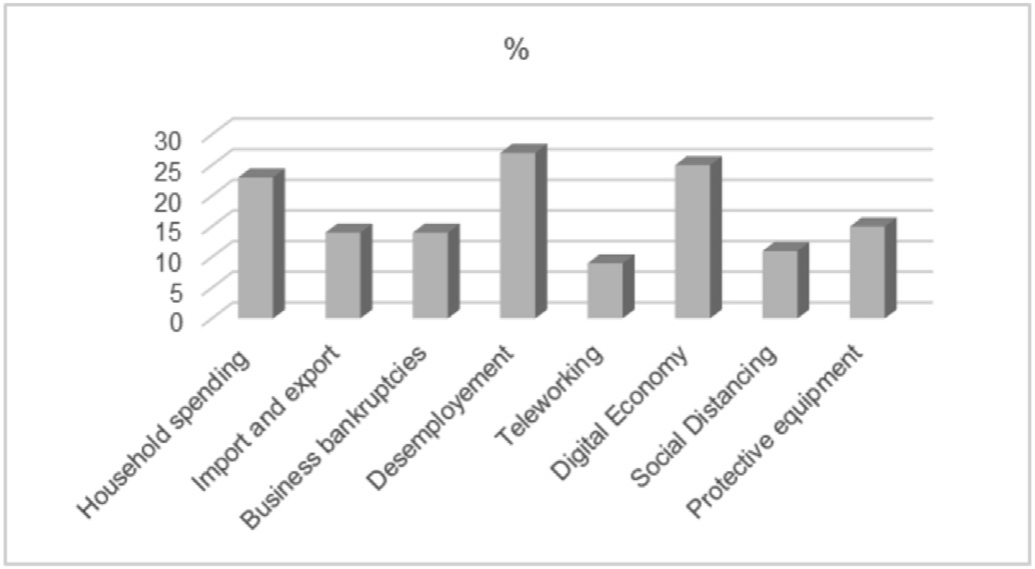
Total of Shocks disrupting disaggregation.

Focusing on shocks derived from the outbreak of COVID-19 on the economy also agreed with the trending topic for self-employed entrepreneurs and companies. The content generated is largely related to unemployment, the digital economy –due to required digital transformation, household spending and the protective equipment to take over business activity and, consequently, to make consumption possible (Figure 4).

**Figure 5:**
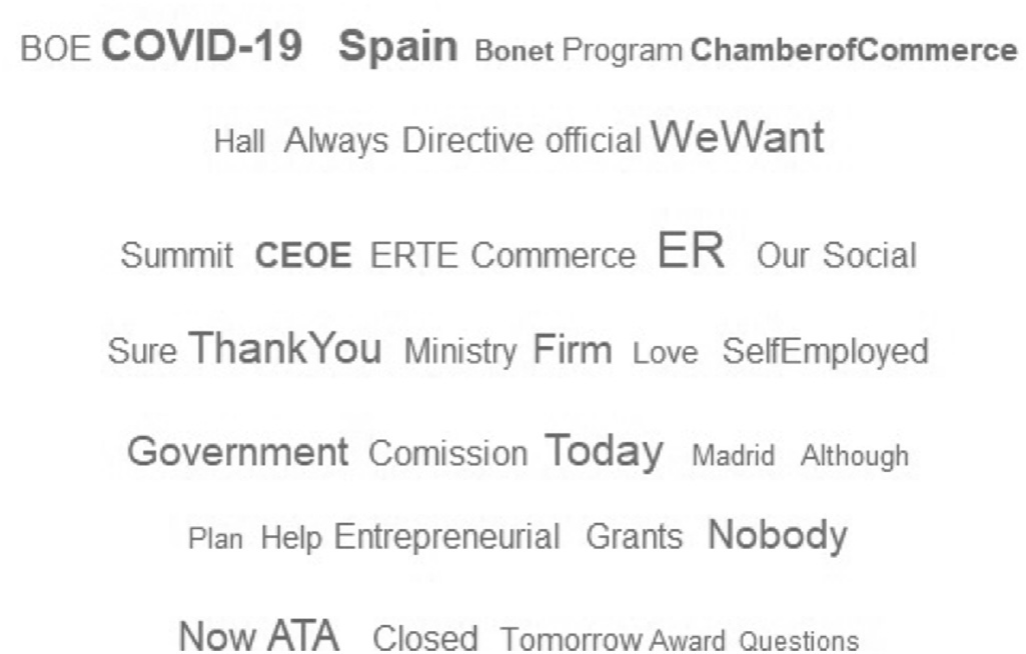
Word cloud.

Regarding the content of the tweets, it should be emphasized that there is balance in the presence of the most highlighted words establishing three levels as shown in Figure 5. The first one with words such as: COVID-19, Spain, Government, Thankyou, Labour Regulation and We want. Only one of the names of the institutions appears in this first level: ATA. The second level includes words such as BOE, grants, companies, self-employed, programs, help, commitment, collective dismissal of workers, directive and plan. All of these are related to legislation/measurements and solutions or actions and, additionally about the main target of these Twitter accounts: business both firms and self-employed. Likewise, the name of the other two institutions appeared in this level: Chamber of Commerce and CEOE. Finally, the presence of two adverbs of time (today and tomorrow) deserves to be mentioned. The uncertainty and the continuous change during the pandemic led to <today> having the leading role in the first level and, consequently, it positioned < tomorrow> in the second level in the word cloud.

**Figure 6:**
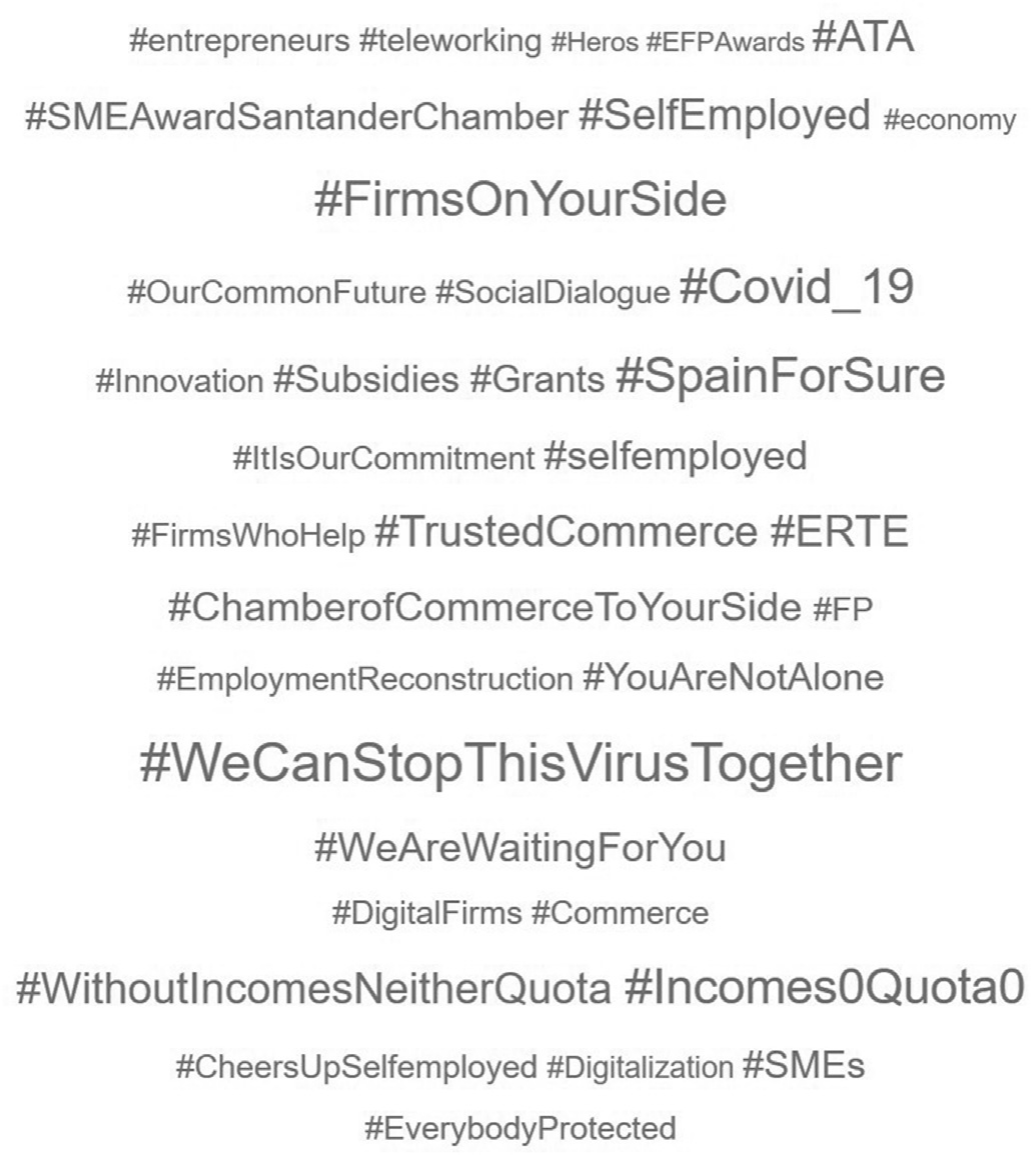
Hashtag cloud.

Conversely, Figure 6 is the resulting word cloud of Hashtags in the three Twitter accounts. It allows comparison of the highlighted issues between waves. There is a higher number of hashtags linked to state of alarm, isolation measurements –especially related to staying at home and messages of encouragement like #EverythingFinish, #EverythingWillBeFine, #CheerupEntrepreneurs, in the first waves. Likewise, the hashtags related to shock that caused the interruption of economic activity and, consequently, the incomes of business #incomes0contributions0 or #withoutincomesnorselfemployedquota should be stressed. In both the first and second analysed waves, the hashtags related to acknowledgements or highlighting the role of health staff like #heroes #careproviders should be noted. From the second waves hashtags about the measures started to appear: #masks, #teleworking #DigitalBusiness. Furthermore, there were even some solutions and requests to carry out firms' activities like #ThisCan'tStop, which contrasted with hashtags that warn about the danger of the disease and the necessary contribution of acting together: #WeStopThisVirusTogether, #SlowDowntheCurve, #ItIsOurCommitment. When the outbreak unfolded, a great deal of topics related directly to the economic and social impact of COVID-19 appeared. Then, the collective dismissal of workers became increasingly important in the third analysed period, while it highlighted the requirement of support for entrepreneurs, so the following hashtags are especially significant, #Social Dialogue and #Help #Reactivation in the fourth waves of the research work. The last two periods analysed alluded particularly to companies: #SMEs, #Firms, #Self-employed and placed value on economic reactivation and confidence building for consumption with hashtags such as: #WeAreWaitingForYou from the fourth wave and, this continued during the fifth wave. The amount of hashtags highlighted an entrepreneurial collective: #firms, #selfemployed, #SMEs and the value of economic recuperation for the benefit of both companies and society was recognized #WeAreWaitingForYou, #Trusted Commerce, #AllProtected or, #SpainforSure. Curiously, both word cloud and hashtags did not refer directly to the “new normal” despite the fact that the content of tweets talked about it without actually naming it.

**Figure 7:**
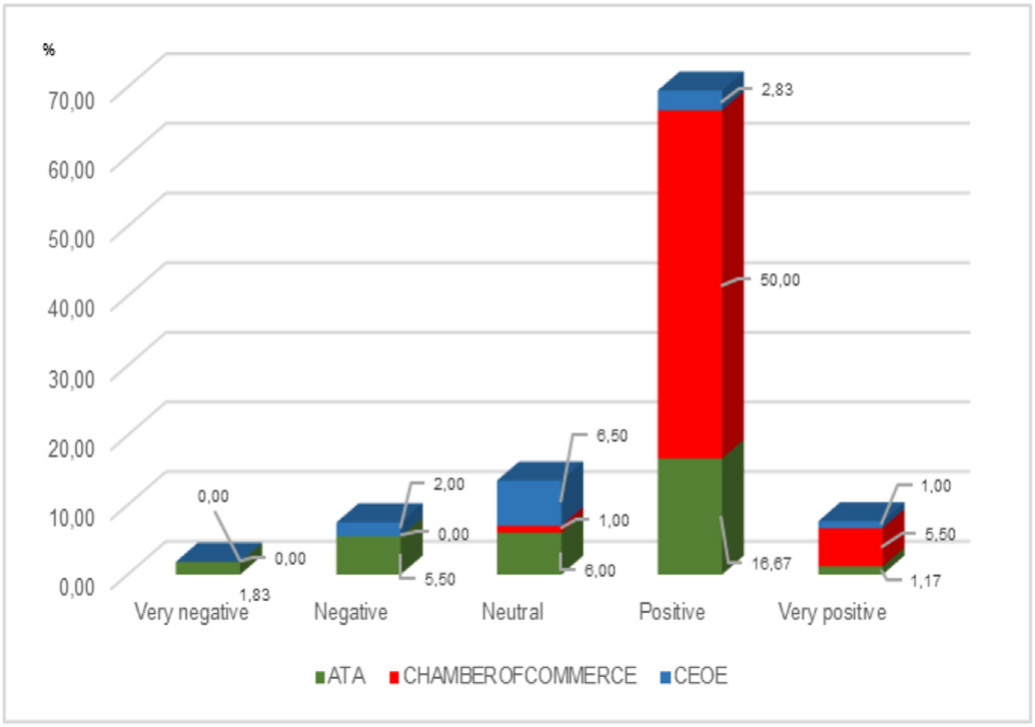
Tweets disaggregated by tone.

**Figure 8:**
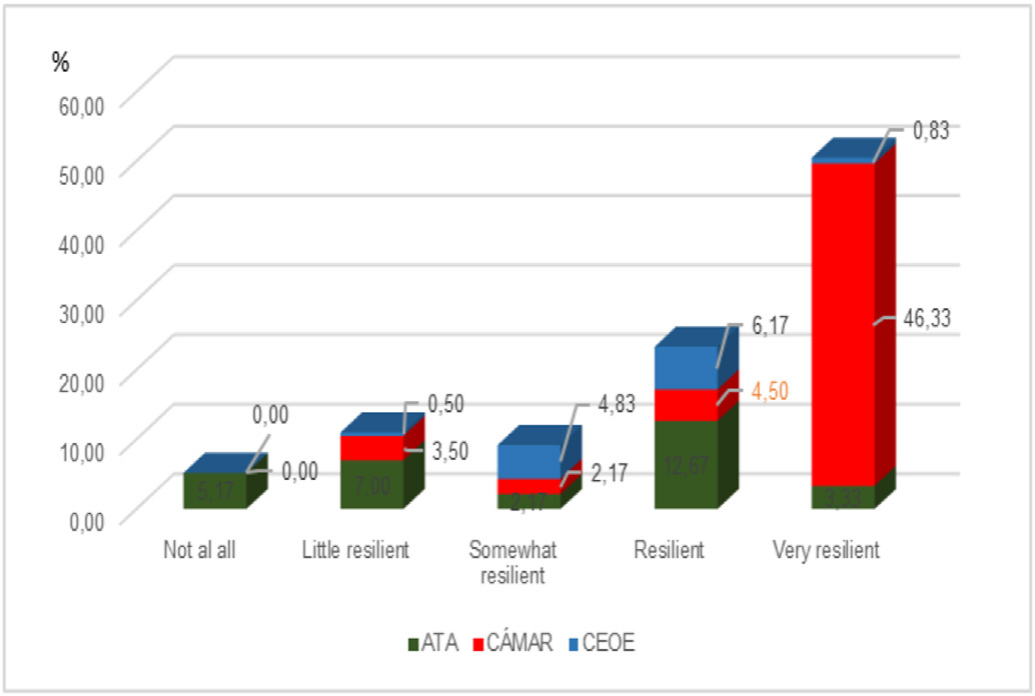
Tweets by level of resilience.

The text corpus of tweets has been analysis according to their tone and it is particularly noteworthy that most of them were characterized as <positive> (Figure 7). The situations derived from the health crisis are really negative but the messages conveyed “hope”. However, all institutions share a positive tone and there are differences in the number of negative or very negative posts. @autonomosata had 24% of content with a negative tone or very negative while in the case of @camarasdecomercio no posts were published without a positive tone. Furthermore, it only had 1.7% with a neutral tone. Regarding CEOE, the negative tone in messages was scarce, 0% very negative and 16% negative vs 31% positive or very positive. This emphasizes that CEOE was the institution, which had more than 50% of its posts associated with a neutral tone. These trends in their publications had a direct relationship with the editorial line in their Twitter accounts. ATA took a more critical view and concentrated a larger number of posts with urgent requirements to the government and tried to show that the situation for the self-employed was more delicate in comparison with that of companies (see Figure 8).

**Figure 9:**
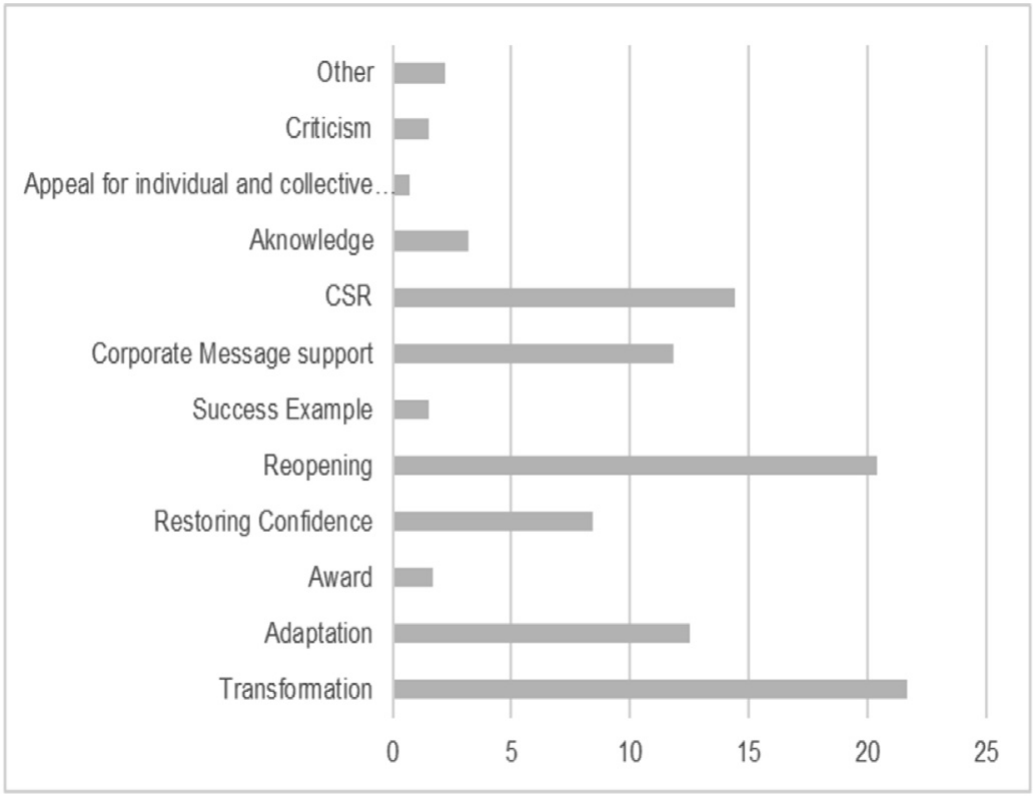
Distribution of tweets according to their main theme.

The level of resilience is consistent with the previous analysis of tone. The higher level in the scale (very resilient and resilient) were the most used (Figure 9). In fact, they represented 74% of the content. However, it is possible to order the institutions according to their level of resilient messages. The Spanish Chamber of Commerce was the most resilient against ATA, which concentrated the less resilient messages. In line with the representation of the abovementioned neutral tone for CEOE, this institution was the organization with greater posts coded as < somewhat resilient>. This is coherent with the typology of content because they use Twitter to inform about legislation, reports and other interesting information for companies. However, @camarasdecomercio mainly focused their efforts on disseminating their programs and projects for companies.

The following provides a holistic vision of the main themes in the tweets analysed (Figure 9). It should be stressed that <transformation>, <reopening> and <adaptation> were the most numerous. In fact, all of them are directly linked with the “new normal” and the changes that companies should assume to continue their activity. Specifically, <transformation > addressed issues related to the most disruptive changes in companies with respect to the situation before COVID-19 and underscoring the digital transformation for business. Meanwhile, <adaptation > especially highlighted social distance, the use of masks and other individual protective equipment. However, this theme was also linked with the requirements of adaptation to the legislation and fiscality to make reactivation and business continuity possible. Finally, <reopening > brought together the posts with regulations during the different phases of the scaling down of the lockdown and proposals that allowed businesses to build trust with their customers. Likewise, the importance attached to corporate social responsibility and corporate messages of support for the collective of each institution are significant in the total number of posts. It emphasizes the tweets whose main topic was restoring people's confidence, mainly customers and consumers and, it is especially noteworthy in relation to commerces. Corporate message support was by its weightiness the next main theme in analysed tweets. The organizations engaged with their target through encouraging messages that also showed their support to entrepreneurs and highlight their role and utility as social agents. It can be illustrated with some hashtags or written content such as #CheerupSelfEmployed, #ChamberofCommerceToYourSide, *the chamber force more than ever, we are all in the same boat* or a *great job.* Additionally, the presence of <restoring confidence> in two directions should be emphasized: for consumers and for promoting companies well disposed to face the new framework where they had to carry out their activity. Finally, it is also worth mentioning the number of messages related to acknowledgement, not only for care providers during the first peak of the pandemic but for entrepreneurs in general or economic activities with a special role in the outbreak, such as supermarkets or transportation of first need goods. Likewise, awards and examples of success to recognize the labour of the business sector and even to provide inspiration to retrain and adapt their activities in the so-called ¨new normal¨ .

**Figure 10:**
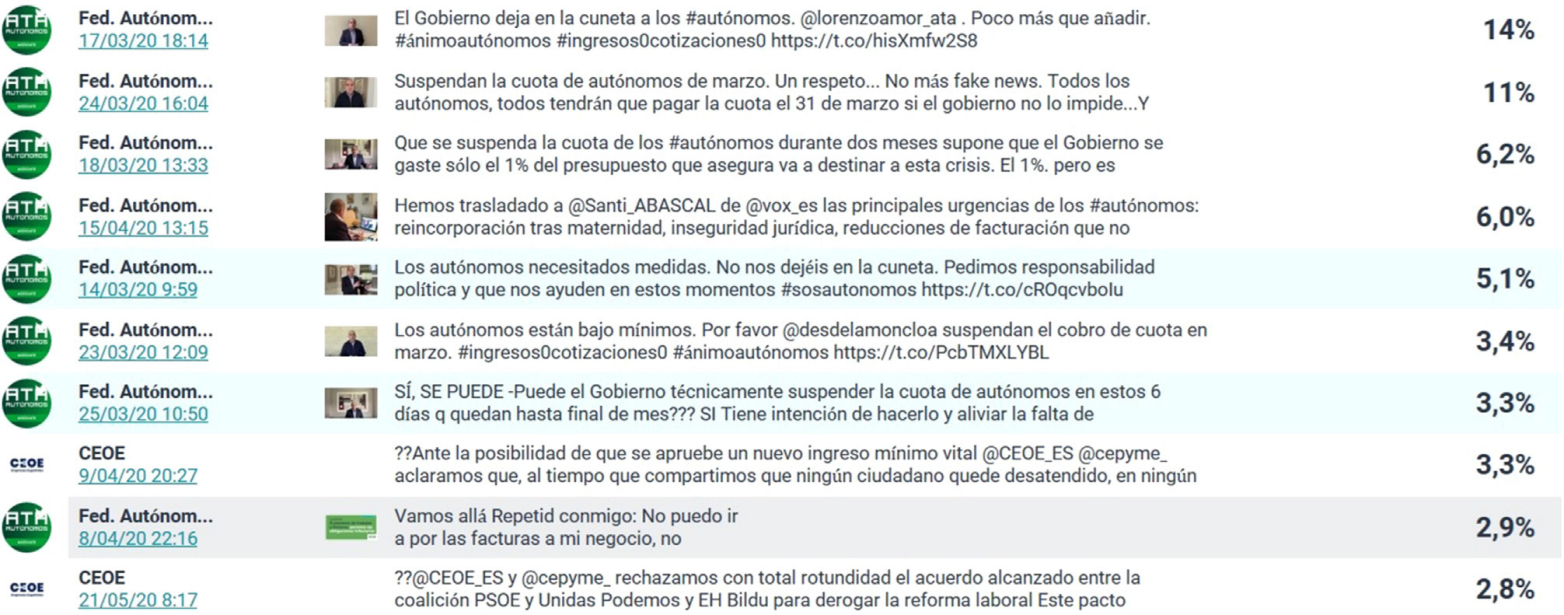
The most outstanding posts because of their interactions.

Following on from the analysis, we can now focus on the interactions and engagement of content generated by these three entrepreneurial organizations. Curiously, it is not the organization with greater publications (@camarasdecomerio) which achieved the highest level of likes and comments. By contrast, @autonomosata was the first in the ranking both in reach and interactions but precisely with the most critical post. It is suggested that the most dramatic situations for the self-employed encouraged discussions and promoted getting requests proposed to the government. In the same line, the highlighted post of CEOE regarding likes and comments also had a critical tone and included a specific request to the government. Moreover, a more detailed analysis of the tweets published by this private entrepreneurial organization showed similar behaviour in the number of likes and comments. The informative posts and content related to activities and programs developed by it got less interaction than those that were more demanding (Figure 10). The Spanish Chamber of Commerce had more a neutral and flatter tone and, consequently, it got fewer reactions and even the increase of followers is less significant than the other two.

In this connection, a correlation analysis was carried out to test if the tone and the level of resilience had a positive impact on the likes of the posts. According to the Pearson correlation, it is significant at the level of 0.01 between likes and tone and at the level of 0.05 between resilience and likes. However, the positive tone reached less volume of likes than negative and very negative tones. The trend was the opposite regarding resilience. In fact, the very resilient posts and the less resilient ones got more likes than those of intermediate positions in the scale.

## 5 Discussion

Uncertainty directly linked with the COVID-19 outbreak impacts on entrepreneurial concerns –expressed through organizations that represent them-about the economic effects on both their own activity and performance and on the economy in general terms. This issue has been pointed out previously for crises in general (Prat and Strömberg, 2013) and those more recently focused on COVID-19 (Baker et al., 2020; Buckman et al., 2020). In line with Wren-Lewis (2020) the weight of this collective perception hampers the reactivation and firms’ adaptation. However, this research work evidences that the three most representative entrepreneurial organizations try to balance the key information about the pandemic (laws, new measures, requirements during the deescalating process etc) with an active reaction. Some of these from a critical point of view and demanding solutions to specific problems and with concise proposals, such as ATA and CEOE while others, such as the Spanish Chamber of Commerce maintain an editorial line which is more focused on programs designed for facing some of the priorities of firms: protective equipment, “trust marks” for comercial businesses etc. The messages on Twitter from either of the versions often chose a positive tone and were generally written with a resilience level. According to Hassan et al. (2020) corporate resilience is identified as direct response to the outbreak and, as the study shows, in a certain way it can be defined as a defense mechanism to move to action for firms and the self employed. This need becomes increasingly important for SMEs and the self employed and the most damaged sectors. The content generated reflects this trend and agreed with Sułkowski et al. (Sułkowski, 2020) who stress the importance of taking them into account, especially to overcome the economic crisis derived from the global health alert. Therefore, there is a common concern about restarting economic activity and working in the “new normal”. This idea is also emphasized by Gourichas (Gourinchas, 2020). The tweets show that organizations are aware of costs and risks for the pandemic. However, they identify hope and are well-disposed to carry out their activity despite the adverse circumstances. Spanish firms and the self-employed feel that they are part of the solution and that they need quick response and radical transformation to survive. The ideas discussed above reveal that there is a collective and main concern with a high burden charge about the future of economic activity, reopening scenarios and strategies to succeed in the “new normal”, consequently, research question 1 finds an answer. Therefore, they generate a feeling of urgency within Spanish business to react to the outbreak with strategies and requirements. Additionally, a sentiment of group and the awareness of join to achieve the needed response is reflected in official Twitter accounts, especially regarding ATA and CEOE. Somehow these findings support the conclusions provided by Pandey and Gupta (2008) and Eldor (2020) about the capability of entrepreneurial organizations to transform and raise collective action because they are the sum of concerns, aspirations of their members as speaker or lobby for them.

Another highlighted issue is foreseeing opportunities to improve their business, for example regarding digital and technology challenges. It is significant in all the posts of every organization. Moreover, successful cases encourage and inspire others to deal with the coronavirus outbreak. From a different point of view but with similar conclusions that have been gathered by Hassan et al. (2020). Thus, it allows us to answer the second research questions. Firms and the self employed share the vision that they are in a really delicate and difficult situation but, at the same time, they become aware of reacting to challenges of the “new normal” being the way. They demand support but from tweets it is perceived that their responsiveness is decisive to achieving it despite the uncertainty. Conversely, this study draws the same conclusion as Samuel et al. (2020) and Abd-Alrazaq et al. (2020) with respect to COVID-19 which means complex challenges and adverse circumstances. However, these authors affirm that the sentiment of American users, are result of digital conversation on Twitter. In fact, this issue is one of the most different regarding existing literature. Finally, there is not a vision of losers and winners as proposed by Hassan et al. (2020). The entrepreneurial organizations and their target assume the situation and know they can not stop. Consequently, the transformation and adaptation were the most referred to topic through social media content.

Furthermore, it should be emphasized that all the organizations play an influent role for their target during the analysed period but the engagement and the interaction are surprisingly higher for the most controversial points and, precisely, the organizations who adopt a more critical editorial line got a higher number of new adepts (followers). As a result, some of the most outstanding posts by their interactivity were themes that especially provoked anger.

The social media coverage of the economic impact of COVID-19 was a recurring theme, in fact, a large proportion of the tweets have been holding this issue. However, there are main topic groups: shock of the production and demand and the proposals made and developed to alleviate the negative effect of the pandemic in business performance and activity. In any event, these topics can be considered the cause of the post and they appear indirectly but the core content is not the analysis of economic impact due to COVID-19 but the call for actions or information about adaptation in order to continue the activity. The topics highlighted in this study are similar to those pointed out by Hassan et al. (2020) for disrupting shocks and by Baker et al. (2020) for the forward-looking measures. Moreover, it should be noted that, when considering Twitter hashtags, some of the most widely used by the three entrepreneurial organizations were with the intention of urging firms to adapt to the new normal (#Can'tStop #Reactivation) proposing specific action as a stamp of safe commerce #Trusted Commerce, empathising with difficult situations experienced (#incomes0quota0) and encouraging all entrepreneurs through unifying messages (#CheersupSelfemployed, #WeCanStoptheVirusTogether). It should be emphasized that hashtags were related to building up consumer/investor confidence (#WeAreWaitingForYou or #SpainforSure).

The analysis of content in the different key periods of pandemic from the entrepreneurial perspective, provides valuable insights into what the main concerns are, reactions, requests and sentiments and how they affected the action developed by the most representative organizations of entrepreneurs in Spain. Previous literature had addressed this issue by focusing on the economical impact of crisis (Eriksson, 2018) and particularly in this unprecedent pandemic (Atkeson, 2020; Baker et al., 2020; Baldwing and Weber Di Mauro, 2020; Bartik et al., 2020) or analysing how Public Relations through social media impacts on health crisis management (Roberts and Veil, 2016; Rodin et al., 2019). Furthermore, there is a growing body of work that pays attention to online conversations linked with sentiments and concerns (Abd-Alrazaq et al., 2020; Buckman et al., 2020; de las Heras-Pedrosa et al., 2020a, b; Fetzer et al., 2020; Samuel et al., 2020) but from the point of view of society. As commented on above, a certain parallelism can also be found between the most referred to topics for citizens and entrepreneurs: unemployment, problems in supply, reactivation measures are common for both (Buckman et al., 2020; Fetzer et al., 2020; Samuel et al., 2020) while the emphasis on different nuances derived from the most strict period of isolation are significant. On one hand, its highlighted society's concern about staying at home (Iglesias-Sánchez et al., 2020) and the figures of contagion and deaths (de las Heras-Pedrosa et al., 2020a, b). On the other hand, taking into account the results of this study, the business sector insisted on those topics about the disruptive effects on their activity, especially regarding performance and staff. The result of this study sheds some light on how the COVID-19 pandemic has gone from being a health emergency to becoming a different world compared to the one that existed before the crisis, as Donthu and Gustafsson (2020 highlight. Consequently, this allows for an analysis of companies'directions to face this new reality on the basis of the suggestions of business organizations. To sum up, this research work contributes to crisis management, emphasizing the importance of taking into account the different stakeholders, in this case, the business sector. In fact, the economic impact of COVID-19 is undeniable but the feelings and predisposition to overcome it from companies and the self-employed could be decisive for an effective “new normal”.

This research is limited to the analysis of communications in the official Twitter accounts of three organizations representative of Spanish business; therefore, we consider that further studies comparing other sub-arenas, such as Instagram, Facebook, etc., other countries, even to focus on content generated directly by firms could deepen the understanding of this phenomenon and how it impacts on strategies and solutions developed. Additionally, this analysis is framed in a national context and in a temporal framework that requires the designing of successive waves of analysis to indentify the real assumption of the “new normal”. Likewise, the monitoring of the practical implementation of proposals made by business organizations in the corporate sector were not subject to the present investigation but would be key for future research work.

Finally, this also entails practical implications for both politicals leaders and for entrepreneurs in order to overcome this crisis. An effort to encourage, share main concerns, empathize and search for solutions, even in a digital environment, can suppose a direct response to the outbreak in order to mitigate and reinvent business. This study warns about the influence on collective concerns and actions for the business community, given that entrepreneurs feel represented by these organizations, they even voluntarily form part of them. Consequently, these organizations should strengthen their social media management as a channel of communication, assuming their role as a source of information, reference for moving to action and of encouraging the corporate sector, even more in times of outbreak and they should also understand that 2.0 channels are a chance to listen and actively involve their entrepreneurs’ members.

## 6 Conclusions

The impact on economic activity due to COVID-19 is one main issue for companies and it is sufficiently proven in content generated by the most representative business organisations in Spain. It is shown in both number of posts and the leading role played out related to the crisis. Furthermore, the official Twitter accounts of three chosen institutions not only published official information with measures, legislation related to the state of alarm, but also their own programs, prospects and analysis of current and future frameworks, messages of encouragement and even, proposals or criticism to overcome the situation and adapt themselves to the “new normal”. Consequently, these organizations adopt a common main strategy of communication focused on messages of encouragement and on generating content about project, initiatives and actions to adapt, transform and continue with economic activity despite the pandemic. In spite of the fact that the transformation and adaptation to the outbreak are the key topic, the concept “new normal” is not specifically used. On one hand, the evolution of interest and followers of all Twitter accounts increased during the outbreak, coinciding with some of the most critical moments of decisions with the growth, for example employment regulation measures. However, the text corpus analysed through the tweets highlights that organisations preferred to communicate a positive tone and resilient messages. On the other hand, the engagement and reaction to messages is higher for the most discussed and controversial points such as the proposal of the suspension of self-employed quota. The neutral posts had little impact on their targets. The results of this study provide a holistic vision of concerns in the business environment through social media. Therefore, it confirms that this outbreak extends to the health area and it has a deep global impact on others such as economy. Furthermore, it reveals a collective sentiment of uncertainty, need for responses to encourage trust in business and adaptation with digital transformation, measures of protection and social distance and with specific response to blend the negative effect on firms' performance. The findings of this research are useful for business organizations to manage their digital content and actions; and to support the companies' and self employed's sentiments to overcome COVID-19 from an economic point of view.

## Declarations

### Author contribution statement

All authors listed have significantly contributed to the investigation, development and writing of this article.

### Funding statement

This work was supported by the Operative Programme 10.13039/501100002924FEDER Andalucía 2014-2020 under grant number UMA18-FEDERJA-148 (Junta de Andalucía/Universidad de Málaga) and Funding for Open Access Charge: Universidad de Málaga/CBUA

### Data availability statement

Data included in article/supplementary material/referenced in article.

### Declaration of interests statement

The authors declare no conflict of interest.

### Additional information

No additional information is available for this paper.

## Footnotes

1 (date 2020/09/30).

2 (date 2020/09/30).
